# Identification of NPM and non-mass breast cancer based on radiological features and radiomics

**DOI:** 10.3389/fonc.2025.1665427

**Published:** 2025-12-01

**Authors:** Zhen Guo, Zhuonan Wang, Xin Lin, Xingyu Chen, Wei Liu, Rui Yan

**Affiliations:** 1 Medical Imaging Center, Northwest Women’s and Children’s Hospital, Xi’an, China; 2 PET-CT Center, The First Affiliated Hospital of Xi’an Jiaotong University, Xi’an, China; 3 Graduate School of Xi’an Medical University, Xi’an, China; 4 School of Computer Science, Xi’an University of Posts and Telecommunications, Xi’an, China

**Keywords:** breast cancer, radiomics, non-mass breast cancer, non-puerperal mastitis, diagnostic accuracy

## Abstract

**Background:**

Non-mass breast cancer, presenting with calcifications, asymmetric dense shadows, and architectural distortions, is challenging to distinguish from non-puerperal mastitis (NPM) due to radiological similarities on mammography.

**Purpose:**

This study aims to develop a mammographic-based radiomics model to differentiate NPM from non-mass breast cancer, addressing the limitations of subjective BI-RADS assessments that risk misdiagnosis or delayed treatment.

**Methods:**

Mammographic images from 104 patients (44 NPM, 60 non-mass breast cancer), collected from January 2018 to June 2023, were retrospectively analyzed. Two senior breast radiologists independently reviewed images, with disagreements resolved by a more senior radiologist. Regions of interest (ROIs) were manually delineated using 3DSlicer, and 576 radiomic features (shape, first-order, texture) were extracted using PyRadiomics. The Least Absolute Shrinkage and Selection Operator (LASSO) algorithm with 10-fold nested cross-validation selected 6 predictive features, and a support vector machine (SVM) model with a Radial Basis Function kernel was constructed. Performance was evaluated using nested cross-validation, calculating the area under the curve (AUC), accuracy, sensitivity, specificity, positive predictive value (PPV), and negative predictive value (NPV).

**Results:**

Calcification type and asymmetric dense shadows differed significantly between NPM and non-mass breast cancer (P < 0.05). The radiomics model achieved an AUC of 0.844 (95% CI: 0.787–0.904), accuracy of 0.769 (95% CI: 0.735–0.803), sensitivity of 0.883 (95% CI: 0.792–0.974), specificity of 0.678 (95% CI: 0.576–0.779), PPV of 0.784 (95% CI: 0.749–0.819), and NPV of 0.778 (95% CI: 0.662–0.896), compared with radiologists’ BI-RADS assessment (AUC: 0.860, 95% CI: 0.790–0.930; accuracy: 0.856, 95% CI: 0.787–0.923; sensitivity: 0.833, 95% CI: 0.736–0.926; specificity: 0.886, 95% CI: 0.791–0.979; PPV: 0.909, 95% CI: 0.832–0.984; NPV: 0.796, 95% CI: 0.679–0.907).

**Conclusions:**

Radiomics using PyRadiomics-extracted features, LASSO, and SVM provides a robust quantitative tool to differentiate NPM from non-mass breast cancer, enhancing diagnostic precision and clinical decision-making.

## Introduction

Breast cancer remains a leading cause of morbidity and mortality among women worldwide, with approximately 2.3 million new cases reported in 2022, making it the most common cancer in women (1). Non-mass breast cancer, characterized by the absence of a distinct mass and blurred margins on imaging, poses a significant diagnostic challenge due to its overlapping radiological features with non-puerperal mastitis (NPM) (2, 3). These non-mass-like (NML) lesions, which include calcifications, asymmetric dense shadows, and architectural distortions, are frequently encountered in clinical practice but are difficult to differentiate using conventional imaging modalities. Misdiagnosis of NPM as non-mass breast cancer can lead to unnecessary invasive procedures, such as biopsies or surgeries, while overlooking malignant lesions may delay critical treatment, adversely affecting patient outcomes (4–6). Therefore, developing accurate and reliable diagnostic methods to distinguish NPM from non-mass breast cancer is of paramount clinical importance.

Traditional diagnostic approaches, such as mammography, ultrasound, and MRI, rely heavily on radiologists’ subjective interpretation using the Breast Imaging Reporting and Data System (BI-RADS) criteria. However, these methods have significant limitations. Mammography, while widely used due to its low radiation dose, often fails to differentiate NPM from non-mass breast cancer because both conditions may present as asymmetric dense shadows or calcifications (7). Ultrasound offers high sensitivity for detecting NML lesions but lacks specificity, leading to high false-positive rates (8). MRI, despite its excellent sensitivity, shows considerable overlap in imaging features between inflammatory and malignant lesions, resulting in diagnostic ambiguity (9). Moreover, BI-RADS-based assessments suffer from inter-observer variability and limited ability to quantify subtle textural differences, which reduces diagnostic accuracy, particularly for NML lesions (10 11).

Prior studies have explored these challenges, with Tan et al. (7) noting that NPM often presents with asymmetric dense shadows, while Barreau et al. (10) reported suspicious microcalcifications as a hallmark of non-mass breast cancer, such as ductal carcinoma *in situ*. Recent work by Gandomkar et al. (11) highlighted the low specificity of ultrasound for NML lesions (AUC = 0.72), and Han et al. (12) demonstrated that MRI-based texture analysis improved differentiation but was limited by cost and availability. Radiomics, an emerging field, extracts high-throughput quantitative features from images, showing promise in breast lesion differentiation. For instance, Zhou et al. (13) achieved an AUC of 0.85 for NPM versus malignant tumors using ultrasound-based radiomics, while Li et al. (14) reported an AUC of 0.78 for mammographic texture analysis of mass lesions. However, mammographic radiomics for NPM versus non-mass breast cancer remains underexplored, with Peng et al. (15) reporting a lower AUC of 0.60 using less robust feature selection, underscoring the need for advanced methods like LASSO and SVM, as used in our study (16).

Radiomics addresses these challenges by providing quantitative, reproducible features, including first-order statistics, shape, and texture features, which capture subtle patterns not discernible to the human eye (17). This study aims to develop a mammographic-based radiomics model, leveraging features extracted via PyRadiomics and selected through the Least Absolute Shrinkage and Selection Operator (LASSO) algorithm, to distinguish NPM from non-mass breast cancer. By offering a quantitative approach, our work seeks to complement radiologists’ assessments and enhance diagnostic precision in clinical practice.

## Materials and methods

### Study population

Eligible female patients who underwent mammography from January 2018 to June 2023 were retrospectively enrolled. Inclusion criteria: (1) with NML-related signs (calcification, asymmetry, structural distortion) on digital mammography; (2) pathologically confirmed NPM or non-mass breast cancer; and (3) complete clinical and imaging data. This study was approved by the local Ethics Committee.

### Mammography

Mammography was performed using the digital mammography system (Selenia, Hologic, USA), and two body positions were routinely scanned, namely, the standard position of the cranio-caudal (CC) position and the mediolateral-oblique (MLO) position. The mammogram images of 104 patients were reviewed independently by two senior breast radiologists blinded joint to clinical and histopathological information. Gland type: scattered fibroglandular breast and fat glandular breast are classified as non-dense, and unevenly dense breast and dense breast are classified as compact. According to the 5th edition BI-RADS (18), radiologists use features such as calcification, asymmetric dense shadow, and structural distortion to determine a patient’s BI-RADS category. BI-RADS Category 4 is widely used to identify imaging findings that generally require interventional diagnostic procedures, with a malignancy probability ranging from 2% to 95%. It is further subdivided into: (1) Category 4A, with a malignancy probability of 2% to 10%, encompassing lesions that warrant intervention but have a lower likelihood of malignancy; (2) Category 4B, with a malignancy probability of 10% to 50%; and (3) Category 4C, indicating a higher suspicion of malignancy but not as definitive as Category 5, with a malignancy probability of 50% to 95%. Therefore, BI-RADS ≤ 4A is classified as non-puerperal mastitis (NPM), while BI-RADS ≥ 4B is classified as non-mass breast cancer.

### Image segmentation

**Figure 1:**
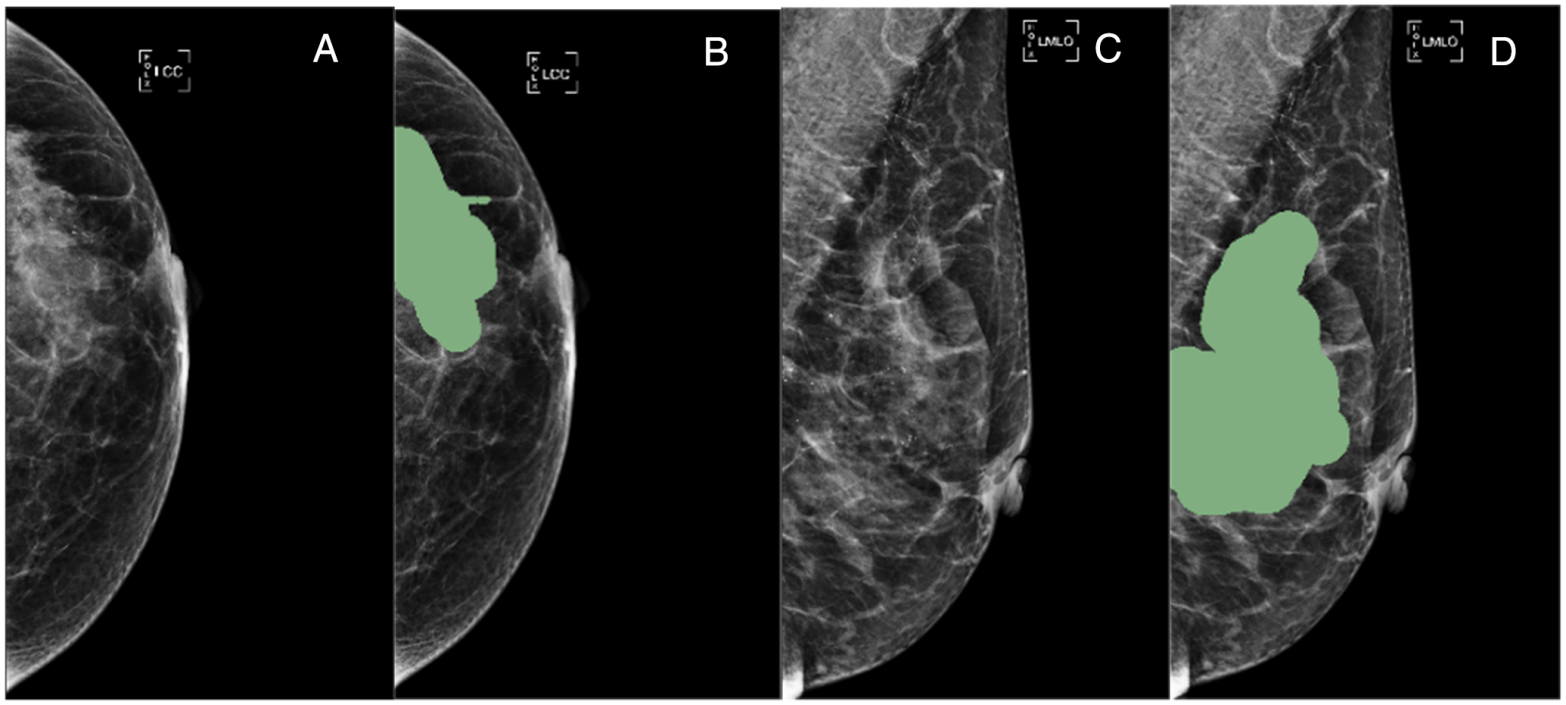
A55-year-old woman presented with carcinoma *in situ* of the left breast duct, BI-RADS 4C. The images showed small pleomorphic calcification in the upper left outer breast quadrant with regional distribution **(A, C)**. The green areas in **(B, D)** showed lesion ROI on the CC and MLO mammograms,respectively. CC, cranio-caudal; MLO, mediolateral-oblique.

Mammogram images, including CC and MLO positions, were loaded into 3DSlicer software (v 4.11, https://download.slicer.org). The region of interest (ROI) of each lesion on two different positions was manually delineated by a radiologist with over 5 years of experience. The significant calcifications in the ROI were labeled. For asymmetrical and structurally distorted lesions, the tagger should delineate the edges where the density of the lesion differs significantly from that of the surrounding glands. Examples are shown in **Figure 1**.

### Feature extraction

Pyradiomics package (v 3.0) for Python software (v 3.7) was used to extract radiomics features. 576 features were retrieved from each ROI, including: (1) shape features; (2) first-order features; (3) second-order texture features, namely gray level co-occurrence matrix (GLCM), gray level run-length matrix (GLRLM), gray level size zone matrix (GLSZM), gray level dependence matrix (GLDM) and neighboring gray-tone difference matrix (NGTDM); and (4) features calculated based on wavelet.

### Feature selection and radiomics model construction

**Figure 2:**
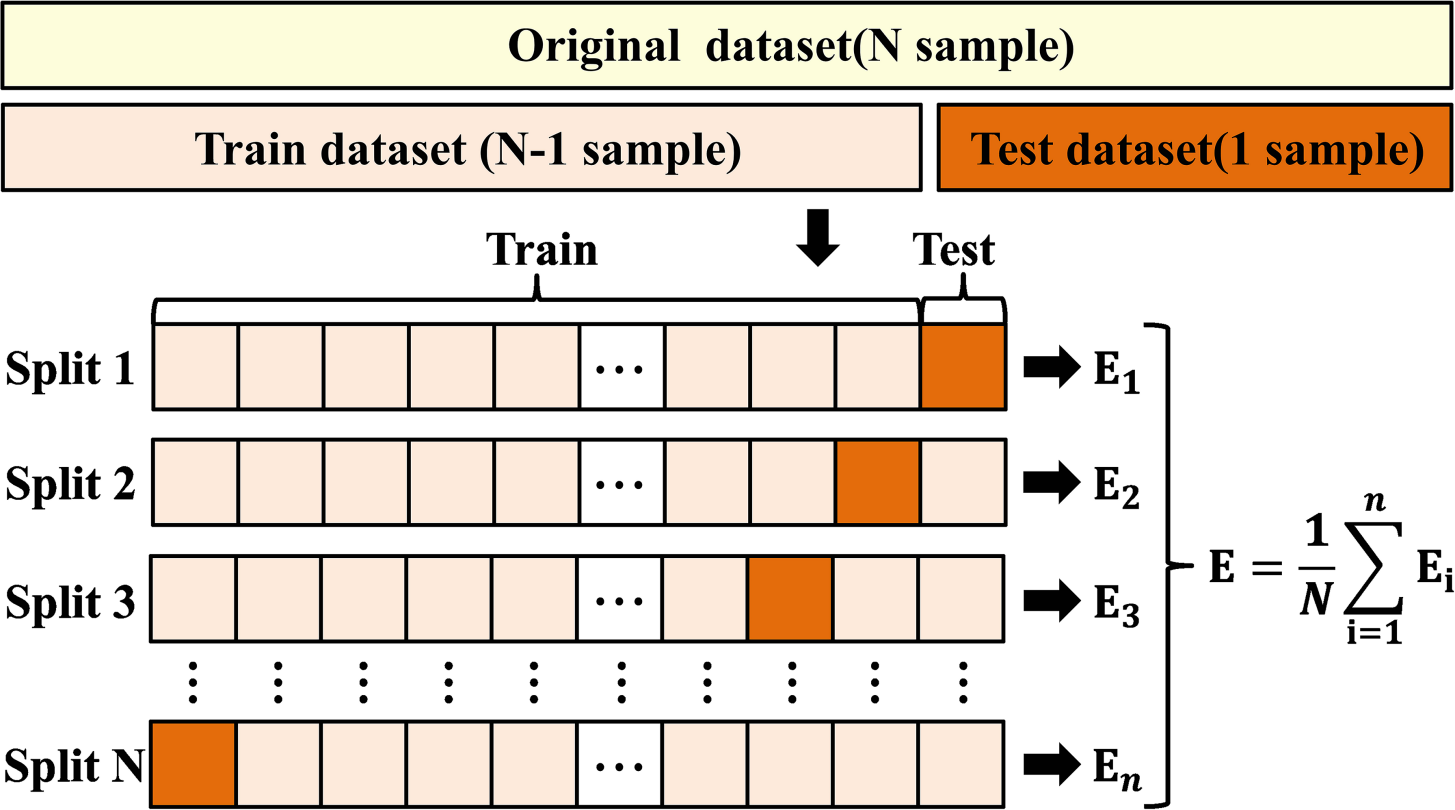
Schematic diagram of the leave-one-out method.

To eliminate index dimension differences, the z-score was used to normalize the retrieved radiomics features before feature selection. The Least Absolute Shrinkage and Selection Operator (LASSO) algorithm was utilized to perform feature selection and calculate the radiomics score for both the training and test sets. LASSO introduces a penalty term (controlled by the regularization parameter λ) into linear regression, shrinking the coefficients of less important features to zero, thus simultaneously achieving variable selection and model compression. To determine the optimal number of predictive features, we applied LASSO with 10-fold nested cross-validation to screen features. The regularization parameter λ was evaluated over a range of (10^-^³, 10^-^¹) using 100 equally spaced values. The model underwent 100,000 iterations of 10-fold nested cross-validation to automatically select the optimal λ value, resulting in λ = 0.0548. At this optimal λ, the 6 features with non-zero coefficients were selected as the most predictive and used to compute the radiomics score. Then, we built a radiomics model using support vector machine (SVM) (**Figure 2**; **Supplementary eTable 1**).

### Statistical analysis

**Figure 3:**
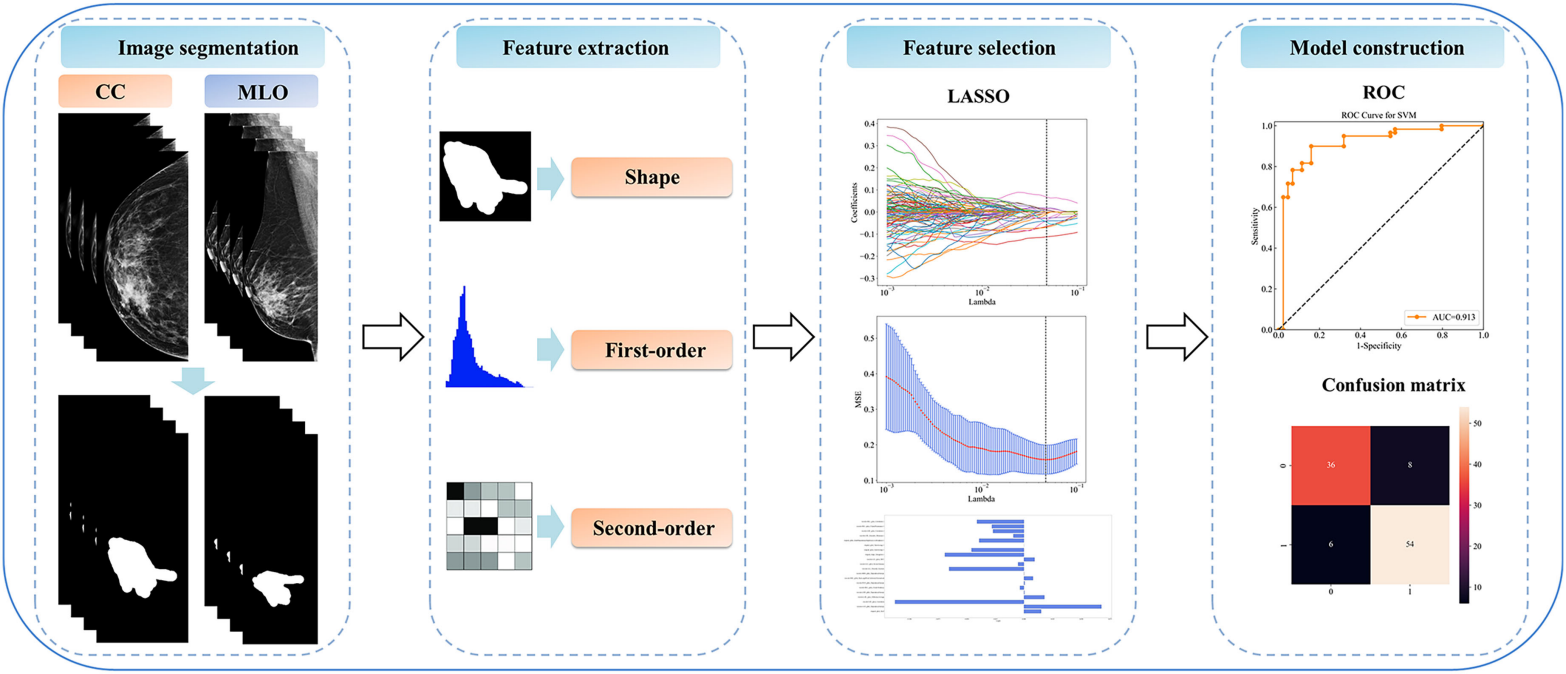
Flowchart of radiomics model construction.

SPSS 27.0 software was used for statistical analysis. The T test and chi-square test were used to compare differences between groups. The Kappa test was used to evaluate the radiologists’ assessment and pathological results. When Kappa is greater than 0.75, it shows a high degree of consistency; when Kappa is between 0.40 and 0.75, it suggests general consistency; and when Kappa is less than 0.40, it indicates poor agreement. The AUC, accuracy, sensitivity, specificity, positive predictive value (PPV), and negative predictive value (NPV) were calculated. The DeLong test was performed to compare the AUC value between the radiomics model and radiologist assessment. P < 0.05 was considered to be statistically significant. The performance of the model was evaluated by the nested cross-validation method. The flowchart of radiomics model construction is shown in **Figure 3**.

## Results

### Clinical characteristics

A total of 104 patients with NML were involved. Of those, 44 (median ± standard deviation: 31.0 ± 4.5 years) with NPM, including acute or chronic mammary inflammation (8/44), suppurative mastitis (11/44), plasma cell mastitis (1/44), granulomatous mastitis (19/44), and granulomatous mastitis with suppurative changes (5/44). And 60 (median ± standard deviation: 46.4 ± 11.3 years) with non-mass breast cancer, including ductal carcinoma *in situ* (35/60), invasive carcinoma (16/60), ductal carcinoma *in situ* with invasive carcinoma (7/60), and ductal carcinoma *in situ* with papillary Paget’s disease (2/60) (**Table 1**).

**Table 1:** Clinical characteristics.

Clinical characteristics of NPM and non-mass breast cancer; Clinical characteristics; Age	NML (n)	
	NPM (44)	non-mass breast cancer (60)
median ± standard deviation(y)	31 ± 4.5	46.4 ± 11.3
pathological types		
acute or chronic mammary inflammation	8	
suppurative mastitis	11	
plasma cell mastitis	1	
granulomatous mastitis	19	
granulomatous mastitis with suppurative changes	5	
ductal carcinoma in situ		35
invasive carcinoma		16
ductal carcinoma *in situ* with invasive carcinoma		7
ductal carcinoma *in situ* with papillary Paget’s disease		2

### Radiologic feature

Among the 44 cases of NPM, 17 cases (38.6%) had Benign calcification, 1 case (2.3%), Malignant calcification, 41 cases (93.2%), Asymmetric dense shadow, and 5 cases (11.4%) of Distorted structure; among 60 cases of non-mass breast cancer, 7 cases (11.7%) had Benign calcification, 50 cases (83.3%), Malignant calcification, and Asymmetric dense shadow. 37 cases (61.7%), Distorted structure 9 cases (15.0%). The relationships between radiological features and pathological types are shown in **Table 2**. There were significant differences in calcification type and asymmetric dense shadow between the NPM and non-mass breast cancer groups (P<0.05, **Table 3**), but no significant differences in gland type and structural distortion between the two groups (P>0.05, **Table 3**). The correlation between radiologists’ assessment and pathological results is summarized in **Table 4**. And the consistency test revealed a general level of agreement between radiologists’ assessment and pathological results (Kappa value 0.679, 0.40 < Kappa value < 0.75). The accuracy, sensitivity, specificity, PPV, and NPV of mammography for differential diagnosis were 0.856, 0.833, 0.886, 0.909, and 0.796, respectively. The AUC for mammography was 0.860 (95% CI: 0.790 - 0.930).

**Table 2:** Relationship between radiological features on mammography and pathological types of NML breast lesions.

Pathological types	Radiological features			
	Benign calcification	Malignant calcification	Asymmetric dense shadow	Distorted structure
Acute or chronic mammary inflammation	3	0	8	0
Suppurative mastitis	6	1	10	2
Plasma cell mastitis	0	0	1	1
Granulomatous mastitis	8	0	18	2
Granulomatous mastitis with suppurative changes	0	0	5	0
Ductal carcinoma in situ	4	30	20	4
Invasive carcinoma	2	12	12	4
Ductal carcinoma *in situ* with invasive carcinoma	1	6	4	1
Ductal carcinoma *in situ* with papillary Paget’s disease	0	2	0	0
Total	24	51	78	14

NML, non-mass breast lesion.

**Table 3:** Analysis of radiological features of NPM and non-mass breast cancer patients.

Radiological features	NPM(n=44)	non-mass breast cancer(n=60)	P
Gland type			
Non-dense	3	7	0.623
Compact	41	53	
Calcification type			
Benign	17	7	0.001
Suspicious	1	50	
none	26	3	
Asymmetric dense shadow			
Yes	42	36	0.001
No	2	24	
Distorted structure			
Yes	5	9	0.591
No	39	51	

NPM, non-puerperal mastitis.

**Table 4:** The correlation between radiologists’ assessment and pathological results.

Pathological results	Radiologists		
	NPM	non-mass breast cancer	Total
NPM	39	5	44
Non-mass breast cancer	10	50	60
Total	49	55	104

NPM, non-puerperal mastitis.

### Model evaluation

**Figure 4:**
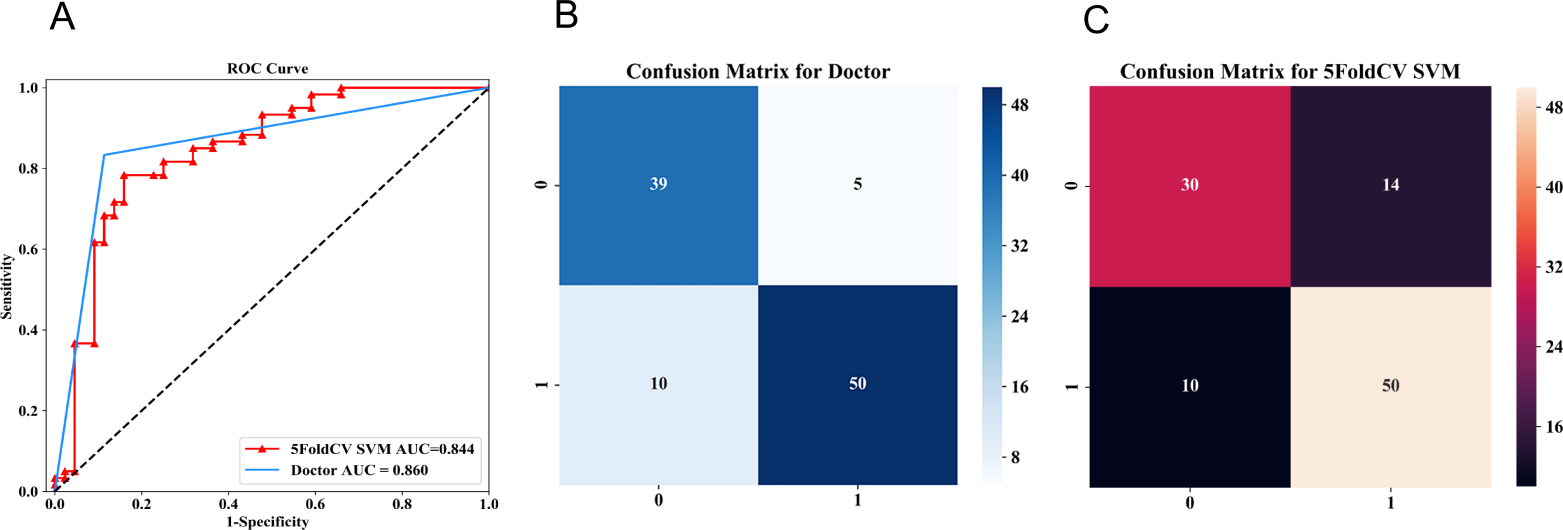
A55-year-old woman presented with carcinoma *in situ* of the left breast duct, BI-RADS 4C. The images showed small pleomorphic calcification in the upper left outer breast quadrant with regional distribution **(A, C)**. The green areas in **(B, D)** showed lesion ROI on the CC and MLO mammograms,respectively. CC, cranio-caudal; MLO, mediolateral-oblique.

Compared with the model without the “age” feature, the AUC value of the model with the “age” feature increased slightly, but the Accuracy, Sensitivity, and NPV decreased, and the Specificity and PPV increased. Six radiomics features were selected as the best features for model building. The accuracy, sensitivity, specificity, PPV, and NPV of the radiomics model were 0.769, 0.883, 0.678, 0.784, and 0.778, respectively. The AUC of the radiomics model was 0.844 (95%CI: 0.787 - 0.904), slightly lower than that of radiologist’ assessment, but the difference is not significant (P > 0.05, **Table 5**, **Figure 4**).

**Table 5:** Diagnostic efficacy of radiologist’ assessment and radiomics model based on mammogram.

Diagnostic efficacy	AUC	Accuracy	Sensitivity	Specificity	PPV	NPV
Radiologists	0.860	0.856	0.833	0.886	0.909	0.796
CI 95%	(0.790–0.930)	(0.787–0.923)	(0.736–0.926)	(0.791–0.979)	(0.832–0.984)	(0.679–0.907)
Radiomics	0.844	0.769	0.883	0.678	0.784	0.778
CI 95%	(0.787–0.904)	(0.735–0.803)	(0.792–0.974)	(0.576–0.779)	(0.749–0.819)	(0.662–0.896)

PPV, positive prediction value; NPV, negative prediction value.

## Discussion

Mammography, as the primary examination for breast diseases, has the advantage of low radiation (19). Radiologists analyzed breast lesions using BI-RADS criteria, but there remained a large number of biopsy-proven benign lesions (20). Lin et al. reported that using BI-RADS as standard, radiologists’ assessment had a sensitivity of 0.863 and a specificity of 0.699 in the diagnosis of breast lesions (21). In this study, the sensitivity and specificity in the differential diagnosis of NML and non-mass breast cancer by using BI-RADS were 0.833 and 0.886, respectively. The consistency test revealed a general level of agreement between radiologists’ assessment and pathological results, implying that using BI-RADS to distinguish NLM from non-mass breast cancer has certain limitations. Our study found that the sensitivity of the radiomics model were higher than that of the assessment by radiologists, indicating that the radiomics can be an effective tool for differentiating NLM from non-mass breast cancer.

The type of calcification can be categorized into typical benign calcification and suspicious calcification. Kim et al. reported that positive findings on mammography in the region of NML, especially suspicious findings such as microcalcifications, can greatly improve the diagnostic accuracy in identifying NML (22). In this study, there was only one suspicious calcification in NPM cases, while there were 50 suspicious calcifications in non-mass breast cancer cases, accounting for 83.3% of the cases, and the type of calcification was statistically different between the two groups (P<0.05), suggesting that the type of calcification could provide a meaningful basis for the detection of non-mass breast cancer. Barreau et al. retrospectively analyzed 909 cases of ductal carcinoma *in situ*, and the most common presentation (75%) was suspicious microcalcifications without associated masses, asymmetric dense shadows or structural distortions (10). Asymmetric dense shadows were statistically different between the two groups of NPM and non-mass breast cancer (P<0.05), which is of great value for differential diagnosis. In this study, it was found that 42 (95.5%) NPM cases presented with the manifestation of asymmetric dense shadows, a finding that is consistent with the findings of the study by Tan et al, who noted that the most common breast X-ray presentation is focal or diffuse asymmetric dense shadow (7).

In this study, LASSO algorithm was used to select 6 radiomics features that were most relevant to the identification of non-mass breast cancer and NPM. Among them, the shape feature - perimeter surface ratio - reflected the irregularity of the border of lesion region by calculating the ratio of the perimeter of the region to the area of the region, and shape feature - elongation - reflected the compactness of the lesion region by calculating the ratio of the long axis length to the short axis length of the region, and shape feature - sphericity - reflected the metric that quantifies how closely the lesion region approximates a circle by calculating the ratio of the square root of the area of the region to the perimeter of the region. The second-order texture features (GLCM, GLDM) quantified the grayscale regions in the image by calculating the grayscale distribution of pixels and their surrounding spatial neighborhoods.

The optimal radiomics features reflect the shape and texture of the lesion region, which could provide quantitative parameters for the differentiation of non-mass breast cancer and mastitis. Li et al. found that texture analysis of mammograms (5 GLCM and 11 GLRLM) could better improve the differential diagnosis efficiency of benign and malignant breast tumors, among which GLCM features have a high weight in distinguishing benign and malignant micro-calcifications (14).

In this study, radiologic features such as calcification type and asymmetric dense shadows were significantly different between NPM and NMBC, echoing previous evidence that suspicious microcalcifications are highly indicative of malignancy whereas asymmetric densities are more often seen in mastitis (7, 10). Texture-based radiomic features selected by LASSO(e.g., GLCM and GLRLM), quantitatively reflect these visual findings. For instance, microcalcifications contribute to heterogeneous pixel intensity patterns, which are captured by higher-order texture features, while asymmetric dense shadows correspond to elongated or irregular lesion morphology, represented by shape features such as elongation (14). Thus, radiomics can be viewed as an objective quantification of radiologists’ visual impressions, and the observed correlation between radiologic and radiomic features strengthens the biological plausibility of our model. Moreover, prior work has demonstrated that combining BI-RADS classification with radiomics improves diagnostic performance and reduces inter-observer variability, highlighting the potential of radiomics to complement expert interpretation in clinical decision-making (23).

To position our work within the context of state-of-the-art methods, we compared our mammographic-based radiomics model with recent approaches for differentiating breast lesions. Unlike ultrasound-based radiomics models, such as those by Zhou et al. (13), which achieved an AUC of 0.85 for NPM versus malignant tumors, our model focuses on mammography and achieves a AUC of 0.844 (95% CI: 0.787–0.904) for NPM versus non-mass breast cancer. Compared to Peng et al. (15), who reported an AUC of 0.60 for mammographic radiomics in NML lesion differentiation using the F-test for feature selection, our use of the LASSO algorithm with 10-fold nested cross-validation (λ = 0.0548) and SVM with RBF kernel resulted in superior performance. Additionally, our model demonstrates performance comparable to that of traditional BI-RADS-based radiologist assessments (AUC = 0.860, 95% CI: 0.790–0.930), as indicated by the non-significant DeLong test (P > 0.05), suggesting that quantitative radiomics may offer a viable complement to subjective visual interpretation. Recent deep learning approaches, such as those reviewed by Ghezzo et al. (24), show promise but require large datasets, whereas our SVM-based model is optimized for small-sample, high-dimensional data (104 samples, 576 features), making it more practical for clinical settings with limited data. These comparisons underscore the novelty and efficacy of our approach in addressing the specific challenge of differentiating NPM from non-mass breast cancer using mammographic radiomics.

A recent systemic review reported that the major machine learning classifiers employed for radiomics based on medical images are logistic regression (LR), random forest (RF), and SVM (24). Wang et al. (25) compared the performance of SVM, LR, and RF for differentiating benign and malignant breast lesions, and the results showed that SVM had a higher diagnostic performance (AUC = 0.820). Hu et al. found that SVM could classify lesions on mammograms into normal, benign, and malignant, with an accuracy of 96.76% (26). In our study, SVM was used for model building, and the AUC was 0.844. By contrast, Peng et al. showed low efficacy in the diagnosis of benign and malignant NML lesions by using radiomics combined with mammography, with an AUC of 0.60, which could be caused by the feature selection method (F test) (15). However, the LASSO algorithm used in our study is more suitable for high-dimensional data.

Our study has some limitations. First, due to the retrospective collection of clinical and pathological data, certain selection bias might exist. Second, the ROI delineation in this study was conducted manually, and errors might be unavoidable due to the difficulty in distinguishing pathological and normal tissue. Thus, automatic segmentation techniques will be involved in the future. Third, this is a single-center study with a limited sample size. Subsequent research should include multicenter samples to confirm the utility of the radiomics model applied in this study (10).

The types of calcification and asymmetric dense shadows on mammograms are valuable in distinguishing NPM from non-mass breast cancer. The radiomics model achieved comparable performance to radiologists’ assessment, suggesting that radiomics may serve as a useful complementary tool in differentiating NPM from non-mass breast cancer.

## Data Availability

The original contributions presented in the study are included in the article/supplementary material. Further inquiries can be directed to the corresponding authors.
