# Supplementary material for: Identification of NPM and non-mass breast cancer based on radiological features and radiomics
Source: Front Oncol. 2025 Dec 1;15:1665427. doi: 10.3389/fonc.2025.1665427 (PMC12702729; doi:10.3389/fonc.2025.1665427)
Supplement: Supplementary file 1 [file Table1.docx]

**Supplementary Materials**

eTable 1. Optimal Feature Set Obtained Using the Nested Cross-Validation Framework

| **Position** | **Feature Name** | **Feature Coefficient** |
| --- | --- | --- |
| CC view breast images (n = 2) | original_shape2D_PerimeterSurfaceRatio | 0.0844 |
|  | wavelet-H_glcm_MCC | -0.0673 |
| MLO-view breast images (n = 4) | original_shape2D_Elongation | -0.0262 |
|  | original_shape2D_Sphericity | -0.0262 |
|  | original_gldm_DependenceEntropy | -0.0777 |
|  | wavelet-H_glcm_MCC | -0.0455 |
